# Advancing biomedical technology through multifunctional porphyrin-based MOFs: design principles, applications, and biosafety evaluations

**DOI:** 10.1016/j.mtbio.2026.103025

**Published:** 2026-03-12

**Authors:** Tushuai Li, Wenxue Sun, Hongying Pan, Qi Tang, Lanxin Geng, Jin Liu, Guangfu Liao, Yanhua Zhang

**Affiliations:** a School of Biology and Food Engineering, Suzhou University of Technology, Suzhou, Jiangsu, 215500, China; b Translational Pharmaceutical Laboratory, Jining NO.1 People's Hospital, Shandong First Medical University, Jining, 272000, China; c College of Materials Engineering, Fujian Agriculture and Forestry University, Fuzhou, 350002, China; d Department of Obstetrics and Gynecology, Binhai County People's Hospital, Yancheng, Jiangsu, 224500, China

**Keywords:** Antibacterial therapy, Biosensors, Biosafety and toxicity evaluations, Cancer treatment, Porphyrin-based metal–organic frameworks, Wound healing

## Abstract

Porphyrin-based metal–organic frameworks (MOFs) have a great deal of promise for widespread use in biomedical and nanomedical fields because of their exceptional structural and functional properties, good biosafety and low toxicity, and intrinsic biodegradability. This review systematically outlines the design principles of porphyrin-based MOFs and highlights their latest developments in a variety of biomedical applications, including cancer therapy, antibacterial treatment, wound healing, and biosensors. Subsequently, we critically examine their biosafety and toxicity aspects. Finally, we detail the current state of the art, ongoing challenges, and future prospects for the emerging materials in biomedicine. With continued research efforts, multifunctional porphyrin-based MOFs have great potential for future clinical translation.

## Highlights

• Design principles of porphyrin-based MOFs were exhibited.
• Latest developments of porphyrin-based MOFs for biomedical applications were outlined.
• Biosafety and toxicity evaluations of porphyrin-based MOFs was examined.
• Current challenges and future research directions were proposed.

## Graphical abstract

**Figure:**
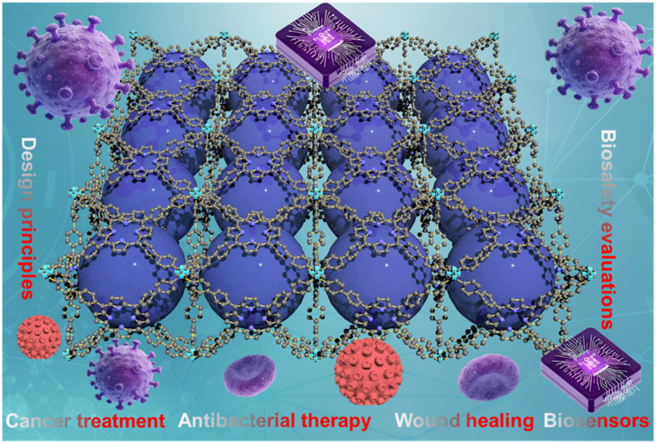

## 1 Introduction

As foundational structures in biology, porphyrins give rise to essential metalloporphyrin complexes such as cytochromes, hemoglobin, and chlorophyll. These ubiquitous “life pigments” fulfill integral roles across diverse living systems [[1], [2], [3], [4]]. The diverse functional capabilities of porphyrins, which include roles in biochemical, enzymatic, and photochemical processes, are largely derived from the singular characteristics inherent to their tetrapyrrole macrocyclic framework [5,6]. The extensive π-aromatic system of porphyrins confers outstanding chemical and thermal stability, along with well-modulated photoelectrochemical characteristics [7]. These characteristics can be finely tuned through variations in the porphyrin's substitution pattern and coordinated metal ions [8]. In addition to metal ion coordination at the center, porphyrins can also bind metals at their periphery [9]. Their complex binding modes allow porphyrins to assemble into advanced molecular cages or framework solids [10]. Moreover, as a major class of organic chromophores, porphyrins and their derivatives demonstrate significant absorption (400-700 nm) at visible wavelengths [11,12]. Thus, porphyrins and their derivatives exhibit diverse uses such as catalysis, solar cell, sensors, and biomedicine because of their unique advantages and versatile functions.

Porphyrin-based metal-organic frameworks (MOFs) are is a new type of porous coordination polymer with two-dimensional (2D) or three-dimensional (3D) topologies, formed through the coordination bonds between metal-containing nodes and porphyrin-based ligands [[13], [14], [15]]. Porphyrin-based MOFs are attracting growing interest because of their exceptional structural and functional features, e.g., ultrahigh porosity, programmable pores, controllable composition, unsaturated metal sites, unique optoelectronic properties, and functional diversity [8]. Moreover, their relatively weak metal-ligand coordination bonds confer intrinsic biodegradability, while the high porosity and large pore dimensions promote favorable loading efficiency for drug molecules [16,17]. These features ensure their potential and promising applications in the field of biomedicine.

In this review, we provide an overview of the latest developments in the biomedical applications of porphyrin-based MOFs, spanning from design principles to biosafety assessment. This review distinguishes itself from existing overviews [8,[18], [19], [20], [21], [22], [23], [24], [25], [26], [27], [28], [29], [30], [31]] by: (i) classifying their design principles based on single porphyrinic MOFs and their composites, (ii) placing particular emphasis on their therapeutic mechanisms, and (iii) underscoring their biosafety features for varied biomedical applications. The review begins with an overview of the design principles for porphyrin-based MOFs. Subsequently, we describe the latest developments in the biomedical applications of porphyrin-based MOFs. Next, we also exhibit the biosafety and toxicity evaluations of porphyrin-based MOFs. Lastly, we discuss the main obstacles and prospects for porphyrin-based MOFs in the biomedical field.

## 2 Design principles for porphyrin-based MOFs

Numerous MOFs, including zeolite imidazolate frameworks (ZIFs) [[32], [33], [34], [35], [36]], Matériaux de l'Institut Lavoisier (MIL) [[37], [38], [39], [40], [41], [42]], and Universitetet i Oslo (UiO) series [[43], [44], [45], [46], [47]], have been developed as versatile platforms for pharmaceutical encapsulation, diagnostic agent delivery, and enzyme immobilization. Historically, the synthetic evolution of porphyrin-based MOFs has adhered to fundamental MOFs design principles [[48], [49], [50], [51], [52]]. For effective biomedical applications, nano-size porphyrin-based MOFs (10-100 nm) becomes imperative due to the intrinsic correlation between particulate dimensions and their physiological behavior post-administration [8,53]. Based on the unique structures formed from porphyrins and MOFs, porphyrin-based MOFs are generally classified as porphyrinic MOFs, porphyrin@MOFs, and porphyrinic MOFs composites. In porphyrin@MOFs systems, unmodified or metal-coordinated porphyrins are incorporated as guest species through pore encapsulation, surface adsorption, or covalent grafting [18]. Conversely, when serving as structural linkers, these porphyrin derivatives directly coordinate with metal nodes or secondary building units (SBUs) to generate porphyrinic MOFs [10]. Porphyrin-based MOFs architectures can be further functionalized through integration with supplementary components such as magnetic nanocrystals, photoconversion materials, and luminescent quantum dots, forming porphyrinic MOFs composites with multimodal capabilities [13]. In the following section, we will briefly describe the synthesis and design methods of porphyrin-based MOFs including porphyrinic MOFs, porphyrin@MOFs, and porphyrinic MOFs composites.

### 2.1 Porphyrinic MOFs

**Fig. 1:**
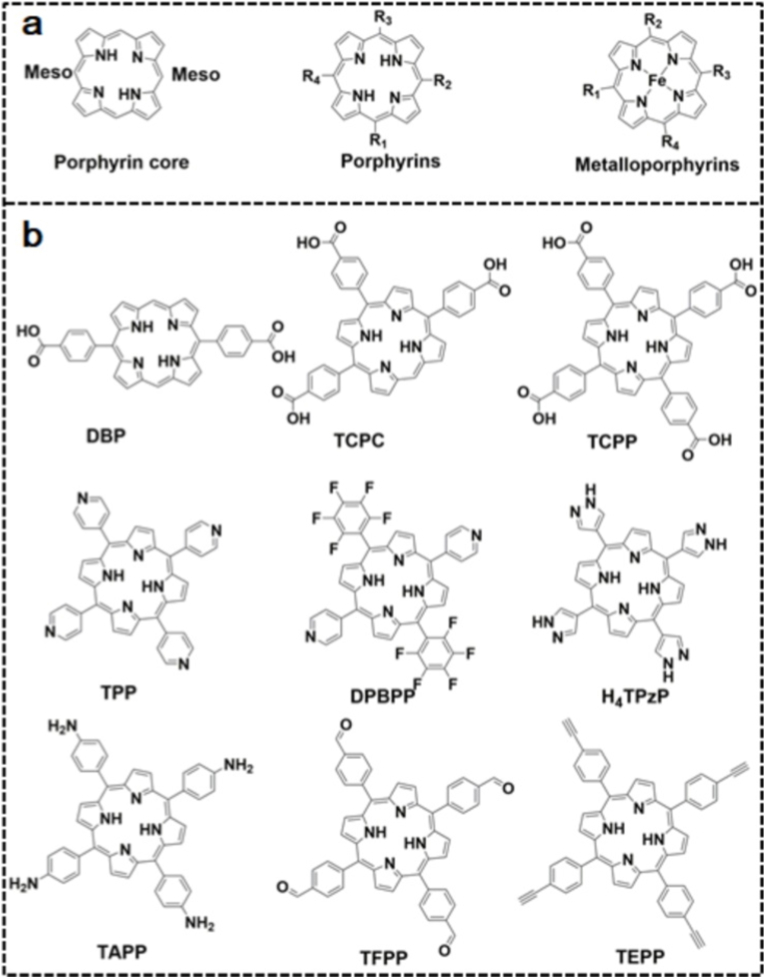
(a) Typical molecular configurations of the porphyrin core, porphyrins, and metalloporphyins. (b) Porphyrin-based ligands for the fabrication of porphyrin-based MOFs.

Porphyrins, as heterocyclic macromolecules, serve as versatile organic linkers capable of coordinating with metal ions or SBUs [54] (Fig. 1a). Such architectures are primarily constructed through the interaction of metallic centers (zinc, copper, iron, etc.) with multidentate ligands (carboxylates, imidazoles, or bipyridyl derivatives), resulting in well-defined polyhedral units. In order to construct the intricate architectures of porphyrinic MOFs, SBUs work as building blocks that assemble into 3D networks using a variety of connecting modes, such as vertex-sharing or edge-sharing [55]. Common SBUs are octahedral structures consisting of four metal ions and six organic linkers, which are represented by the notation [M_4_(O/R)_6_]. SBUs have a substantial impact on the photoelectrochemical performance of porphyrinic MOFs [56,57]. Fig. 1b shows some typical porphyrin linkers. This section systematically elaborates three distinct fabrication methodologies for porphyrinic MOFs: (1) topological, (2) metal-organic cage, and (3) pillar-layer engineering.

#### 2.1.1 Topological engineering

Topology, being foundational in mathematics, investigates how certain spatial properties persist despite continuous deformations [[58], [59], [60], [61]]. Topology fundamentally analyzes how objects maintain connectivity and contain voids, abstracting away from metric properties like shape or size. Chemical topology in synthesis prioritizes molecular linkage patterns and neighbor relationships, treating spatial coordinates as secondary parameters. This method emphasizes the connection between lines, which stand for connections including chemical bonds or edges, and points, which describe entities such as atoms or vertices [62]. Topological descriptor-assisted design of porphyrinic MOFs enables rational prediction of framework architectures and property optimization through quantitative index analysis. By offering direction for structural design and synthesis, topological engineering makes it easier to simplify and analyze intricate porphyrin-based MOF structures. Nowadays, a common topological approach is to preserve the intrinsic topology while building porphyrinic MOFs to guarantee structural soundness and desired performance [[63], [64], [65]]. At the chemistry-biology frontier, topological precision in porphyrinic MOFs directly correlates with enhanced bioactivity metrics, from photosensitizer efficiency to controlled drug release kinetics. For instance, altering the linker length in porphyrinic MOFs can change the pore size [[66], [67], [68]]. Moreover, the physicochemical characteristics of porphyrinic MOFs can be altered by altering the coordinating metal in the porphyrin core [21,69]. In addition, strategic modulation of metal-ligand coordination geometries (bond lengths/angles) via cluster/node engineering affords enhanced structural robustness in porphyrinic frameworks [70,71].

**Fig. 2:**
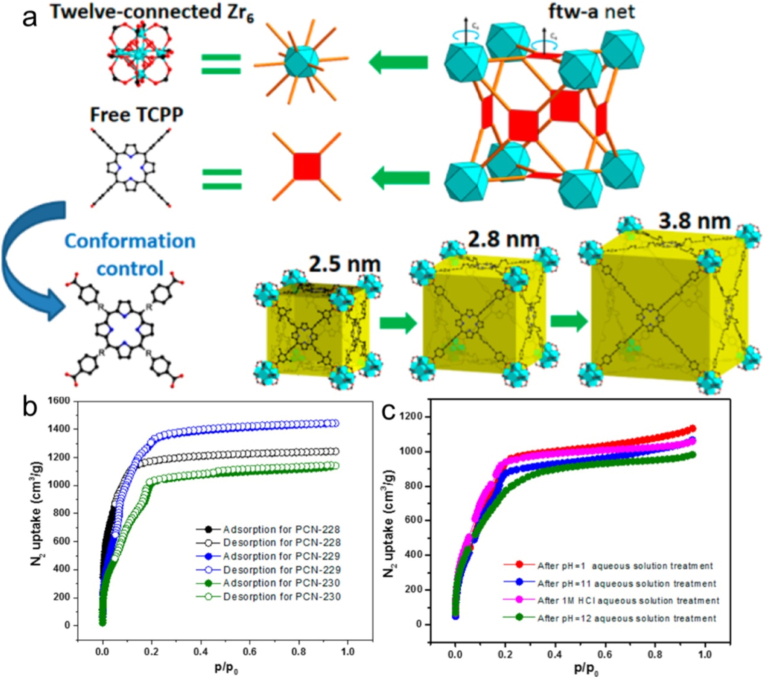
(a) Zr_6_-containing isoreticular porphyrinic MOFs through topological engineering approaches. (b) N_2_ adsorption isotherms of PCN-228, PCN-229, and PCN-230. (c) N_2_ adsorption isotherms of PCN-230 after various treatments. Reproduced from Ref. [72].

Porphyrin is made up of exterior functional groups and a center heterocyclic ring (Fig. 1). It is simple to alter the length and structure of these functional groups. Porphyrin, which is typically big (approximately 2.5 nm), thus serves as a linker that makes it easier for large pores to form inside the organic framework, resulting in the formation of mesoporous porphyrinic MOF. For example, Zhou and co-workers [72] developed various Zr_6_-comprising isoreticular porphyrinic MOFs including PCN-228, PCN-229, and PCN-230, with **ftw-a** topology via topological engineering approaches utilizing prolonged porphyrinic linkers (Fig. 2a). In this work, the carboxylate group and the vicinal phenyl ring were arranged to lengthen the necessary organic linkers. The maximum porosity and BET surface area are found in PCN-229, which has pore apertures ranging from 2.5 to 3.8 nm (Fig. 2b). Notably, material stability exhibits an inverse correlation with linker length. A case in point is PCN-230, which demonstrates remarkable stability in aqueous solutions across the extreme pH range of 0-12, showcasing its exceptional chemical robustness and pH tolerance (Fig. 2c). The tunable linker length in porphyrinic MOFs offers a promising pathway to expand their biomedical applications such as cancer treatment, antibacterial therapy, etc.

**Fig. 3:**
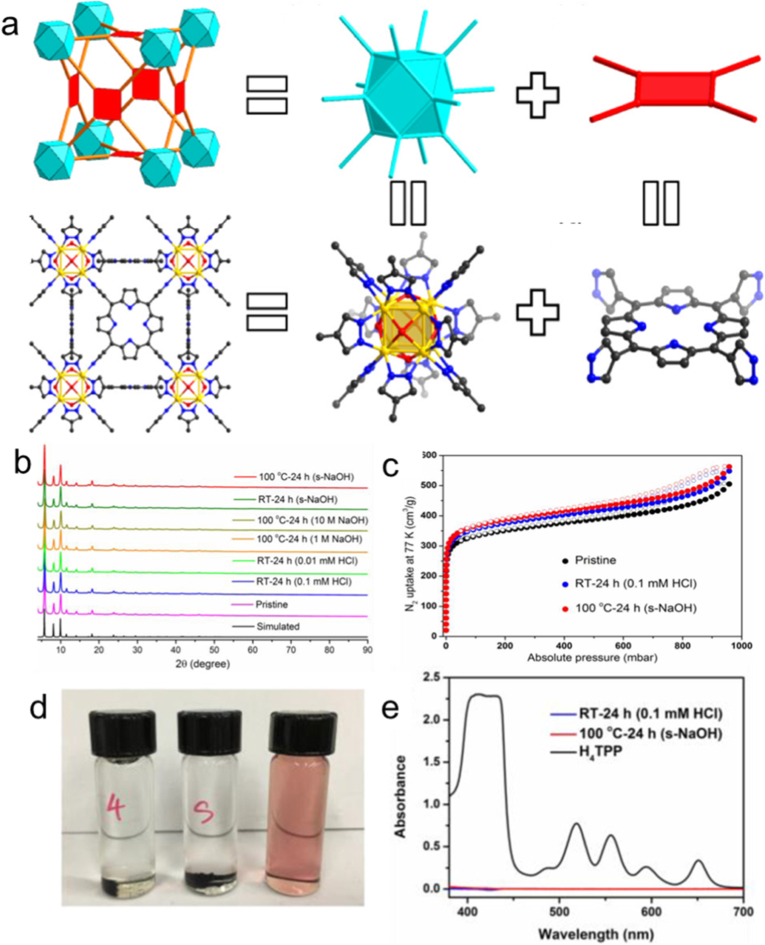
(a) Synthesis strategy for PCN-601. (b) PXRD patterns of PCN-601 under different circumstances. (c) N_2_ adsorption/desorption isotherms of original PCN-601 sample and PCN-601 samples treated with acid and alkali. (d) N,N-Dimethylformamide solutions of immersed PCN-601 samples treated with 0.1 mM HCl solution at room temperature for 24 h (left) and saturated NaOH at 100 °C for 24 h (middle), respectively. And the standard solution of H_4_TPP in N,N-dimethylformamide (right). (e) UV-vis spectra of various N,N-dimethylformamide solutions from the vials in (d). Reproduced from Ref. [77].

Furthermore, the coordination dynamics between metal nodes and organic linkers significantly influence the chemical robustness of porphyrinic MOFs [73]. On the basis of the hard-soft acid-base (HSAB) rule, modulating the electron affinity of metal ions and porphyrin moieties enables precise tuning of the framework's stability [74,75]. For instance, porphyrinic MOFs featuring high-valent metal nodes and carboxylate-functionalized linkers demonstrate pronounced structural adaptability in aqueous environments [76]. According to this principle, Zhou and co-workers [77] developed a new porphyrinic MOF (denoted as PCN-601) with extraordinary base-resistance through a facile top-down approach. The prepared PCN-601 with **ftw-a** topology consisted of the Oh symmetric 12-connected [Ni_8_(OH)_4_(H_2_O)_2_Pz_12_] (namely [Ni_8_]) nodes and the *D*_*4h*_ symmetric 4-connected 5,10,15,20-tetra(1Hpyrazol-4-yl)porphyrin (H_4_TPP) ligands (Fig. 3a). Remarkably, this material maintains both crystallinity and porosity when immersed in concentrated NaOH solutions across a wide temperature range from ambient conditions up to 100 °C (Fig. 3b and c). The exceptional alkali resistance of PCN-601 in aqueous solutions grants it distinctive advantages for biomedical applications, particularly in photodynamic and photothermal therapies, where porphyrinic MOFs often demand robust base tolerance. Therefore, topological engineering is vital in designing porphyrinic MOFs with multi-functionality for promising biomedical applications.

#### 2.1.2 Metal–organic cage engineering

Discrete organic building pieces self-assemble to produce hollow supramolecular structures known as molecular organic cages. These structures arise from directional non-covalent interactions that precisely organize multiple components into three-dimensional frameworks enclosing internal cavities [[78], [79], [80]]. The ability of hollow self-assembled molecular cages to selectively incorporate substances into their pre-existing cages is extraordinary. Hollow supramolecular cages demonstrate exceptional host-guest chemistry capabilities, enabling selective encapsulation of target molecules. These architectures not only provide stabilized storage and controlled transport environments for guests, but also facilitate their catalytic transformation through confined-space effects [81,82]. Su and co-workers [83] reported two very stable porphyrinic MOFs, e.g., ([Zr_6_O_4_(OH)_4_(H_2_TBPP)_3_]_n_ (solvent)_x_) (FJI-H6) and ([Hf_6_O_4_(OH)_4_-(H_2_TBPP)_3_]_n_ (solvent)_x_) (FJI-H7), both featuring 2.5 nm cages. FJI-H6 and FJI-H7 consisted of 12-connected M_6_O_4_(OH)_4_(CO_2_)_12_ nodes (M = Zr, Hf) and porphyrin tetracarboxylic ligands (H_6_TBPP), which exhibited ultra-high specific surface area of 5007 m^2^ g^−1^ and 3831 m^2^ g^−1^, respectively. Moreover, they also exhibited exceptional chemical robustness, maintaining structural integrity across a broad pH range (0-14). Beyond their outstanding acid/alkali resistance, they function as programmable hosts for selective molecular encapsulation, enabling stabilized storage, controlled transport, and confined-space catalysis of guest species. Their unique properties hold significant promise in biomedical applications, such as targeted drug delivery, enzyme mimicry, and bioimaging.

#### 2.1.3 Pillar-layer engineering

The pillar design in MOFs was initially developed by Kitagawa's team, enabling a 3D arrangement through hydrogen-bonded interlayer connections [78]. Through pillar-layer engineering, the 2D structure of porphyrinic MOFs can be straightforwardly and efficiently transformed into a 3D form [84,85]. For example, Zhou and co-workers [86] fabricated Zr-based porphyrinic MOFs with a layer-pillar structure. In this structure, hexagonal 6-coordinated Zr_6_ clusters and triangular BTB linkers are connected to generate a 2D layered (3,6)-connected **kdg** topology. This structure features Zr_6_ metal clusters bonded to six carboxylate groups in the 2D layer, with six pairs of terminal –OH/H_2_O ligands on both sides of the layer to facilitate carboxylate linker attachment. The 2D layered MOFs are transformed into a 3D layer-pillar structure through using ditopic ligands as pillars to link the 2D layers together. Notably, the formation of Zr-COOH bonds is exothermic, which energetically favors porphyrinic MOFs with Zr_6_ nodes. Furthermore, Zhang and co-workers [87] reported two porphyrinic MOFs consisting of 5,10,15,20-tetrakis(4-carboxyphenyl)porphine (TCPP) and 1,2-bis(1*H*-benzo[*d*]imidazole-2-yl)ethene (BIE) conjugated ligands. In both orphyrinic MOFs, Zn_2_(CO_2_)_4_ paddlewheel units were linked through TCPP-Zn ligands to create 2D layers. BIE ligands then connected these layers, forming a 2D bilayer in PMOF-1 and a 3D pillar-layered framework in PMOF-2, serving as conceptual models to evaluate how dimensions affected third-order nonlinear optical performance. The 3D pillar-layered framework in PMOF-2 was found to promote interlayer charge migration more efficiently than the 2D bilayer in PMOF-1, resulting in enhanced third-order nonlinear optical property. Thus, pillar-layer engineering is crucial to adjust and optimize the structure of porphyrinic MOFs, which endow them with unique properties for biomedical application.

### 2.2 Porphyrin@MOFs

**Fig. 4:**
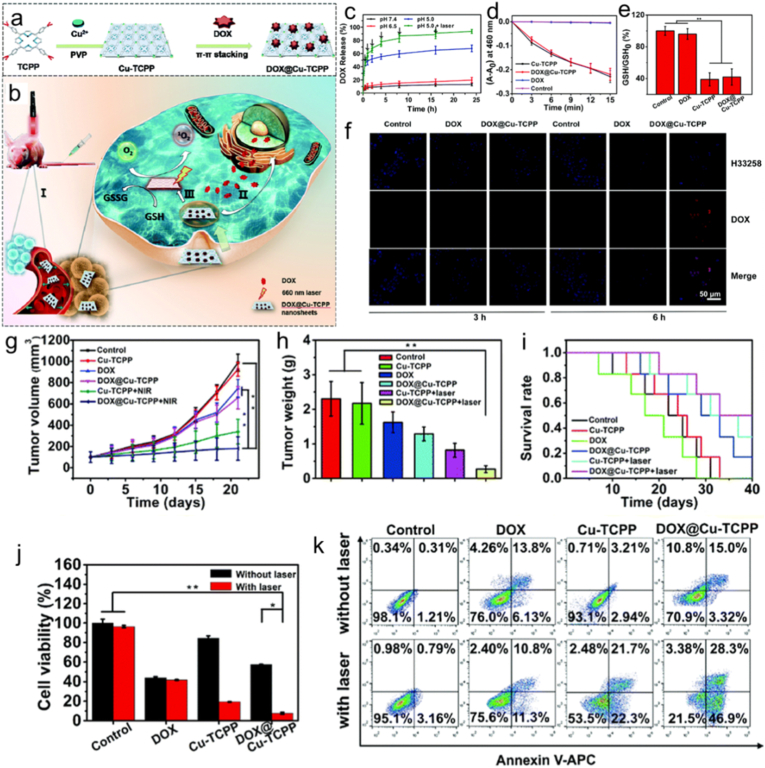
(a) Preparation procedure and (b) anticancer mechanism of DOX@Cu–TCPP nanosheets. (c) pH- and laser-triggered drug release from DOX@Cu–TCPP nanosheets. (d) Time-dependent reactive oxygen species (ROS) production by Cu–TCPP and its nanocomposite. (e) Glutathione (GSH) depletion in various mixtures after 6-h incubation. (f) Confocal imaging of 4T1 cell internalization at 3- and 6-h time points. (g) Tumor volume measurements. (h) Tumor weight quantification. (i) Kaplan–Meier survival analysis of treated mice. (j) Cytotoxicity of DOX, Cu-TCPP, DOX@Cu-TCPP, and a blank control against 4T1 cells under both illuminated and non-illuminated conditions. (k) Quantitative apoptosis analysis using Annexin V-FITC/DAPI staining. Reproduced from Ref. [90].

In order to improve the activity and stability of porphyrinic MOFs, researchers commonly introduce porphyrins into MOFs to develop porphyrin@MOFs [26]. The encapsulation synthesis of porphyrin@MOFs is achieved through a one-pot strategy in mixed solution systems. By integrating porphyrins as guest molecules into MOFs through pore encapsulation, surface adsorption, or grafting interactions, these hybrid materials can be effectively synthesized. Owing to its operational simplicity, time efficiency, and cost-effectiveness, this one-pot approach has garnered significant research interest while opening new avenues for the practical applications of MOF-encapsulated materials [18,88,89]. Recently, Cai and co-workers [90] developed a pH-responsive medication delivery method based on a 2D MOF using a straightforward bottom-up approach (Fig. 4). Through a hydrothermal method, these components self-assembled into copper porphyrinic MOFs (Cu-TCPP) nanosheets. Subsequently, doxorubicin (DOX) was efficiently decorated onto the Cu–TCPP nanosheets via π–π stacking interactions, achieving a remarkable drug loading capacity of 33%. The pore structure and functional groups Cu-TCPP greatly affects the controlled release kinetics of DOX, and thus the resulting DOX@Cu–TCPP nanosheets exhibited pH-triggered drug release properties and remarkable glutathione (GSH) scavenging capability. Moreover, they also produced rich cytotoxic ROS under light illumination due to photosensitive nature and controllable pore structure. Moreover, they also exhibited outstanding anti-tumor efficiency and biocompatibility. Consequently, porphyrin@MOFs has been considered as a pivotal approach to improve anti-tumor efficiency, wherein the judicious selection of porphyrins during synthesis critically governs pore structure, morphology, and dimensions.

### 2.3 Porphyrinic MOFs composites

To endow porphyrinic MOFs with multifunctionality, integrating functional components into their frameworks to form composite materials represents a viable tactic. So far, diverse functional components, i.e., metal nanoparticles [91,92], metal sulfides [93], metal oxides [94], porous polymer [95,96], biomolecules [97], etc have been incorporated into porphyrinic MOFs to produce multifunctional composites for biomedical applications. For instance, core-shell composite porphyrinic MOFs were formed through the growth of porphyrinic MOFs on PDA nanoparticles, GO nanosheets, and Au nanorods. By precisely modulating the coordination interactions between the functional groups and Zr nodes, this strategy effectively suppressed MOF self-nucleation in solution while simultaneously regulating the thickness of porphyrinic MOFs [98]. It has been demonstrated that this porphyrinic MOFs composite has enormous potential in the biomedical area.

**Fig. 5:**
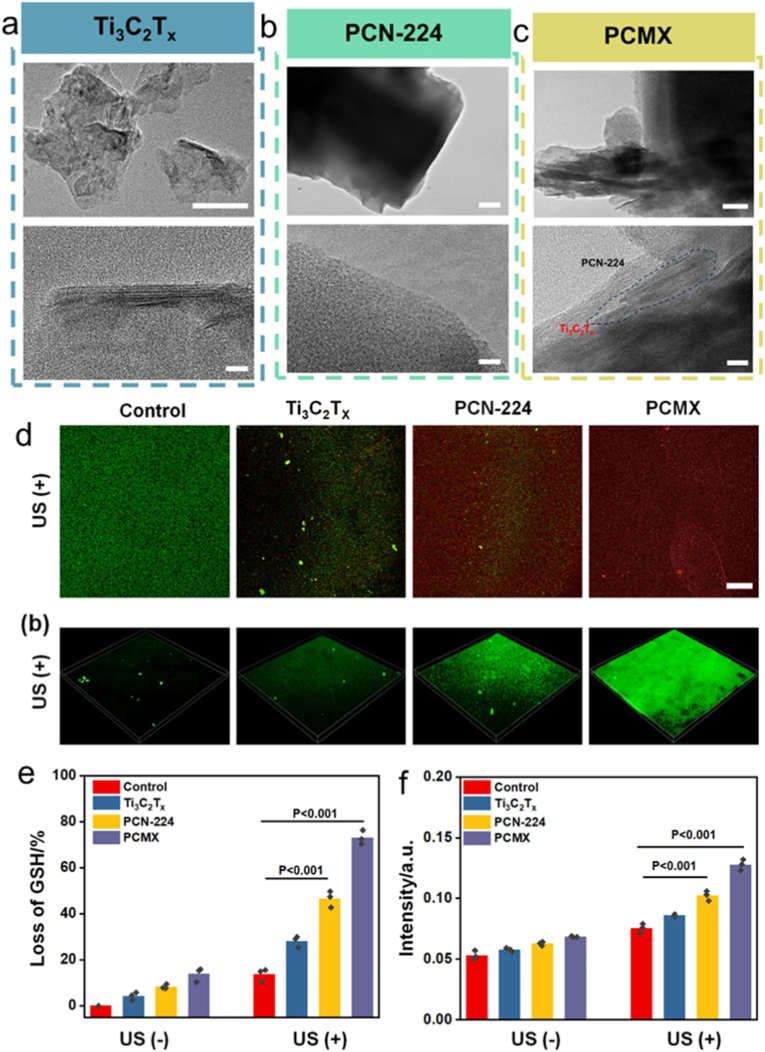
TEM and HRTEM of (a) Ti_3_C_2_T_x_, (b) PCN224, and (c) PCMX. (d) Pictures of internal ROS and live/dead bacteria following various sample treatments. (e) GSH depletion in various samples following US or non-US therapy. (f) Permeation rate of bacterial membranes in various samples following treatment with or without US. Reproduced from Ref. [101].

Surface alterations are necessary to regulate the interface interaction in order to produce porphyrinic MOF composites with controllable shape [19,99,100]. The interactions between porphyrinic MOF and other component possess a significant influence on the structure and performance of porphyrinic MOF composite. A simple hydrothermal method was used to construct an ultrasound-triggered Schottky heterojunction (PCMX) composed of 2D MXene (Ti_3_C_2_T_x_) and porphyrin MOF (Fig. 5a–c) [101]. Under ultrasound irradiation, the prepared PCMX generated carriers efficiently. The Schottky junction in PCMX promoted electron-hole separation and inhibited electron backflow, and thus improving charge utilization and boosting reactive oxygen species (ROS) yield. Based on these advantages, the PCMX showed great potential for photocatalytic antibacterial treatment. Heterojunction building increases the speed of electron transport, opening up a new avenue for the production of highly effective antimicrobial nanomaterials (Fig. 5d–f). The formation of heterojunctions accelerates electron transfer, offering a novel approach to fabricating highly antibacterial nanomaterials. Additionally, a CuNi-MOF-based chitosan-cationic guar gum conductive hydrogel (CHG/CuNi-MOF) was reported as a signal amplifier for trace acetaminophen determination. It exhibited good stability, repeatability, and anti-interference performance, enabling selective target detection in complex samples and achieving satisfactory recoveries [102]. Of course, the porphyrinic MOF heterojunction is also very important for biomedical applications due to their good biocompatibility and outstanding catalytic efficiency [[103], [104], [105]]. For example, Song and co-workers [101] reported a Ti_3_C_2_T_x_-porphyrin MOF heterojunction, which was used for boosting ROS production and antibacterial therapy. The formation of heterojunctions improved the electron migration rate, offering a novel strategy for developing nanomaterials with high antibacterial efficiency.

Porphyrin-based MOFs are more stable and have longer circulation times in biological systems when their surfaces are modified with biocompatible substances such as polyethylene glycol and polyvinyl pyrrolidone. Table 1 provides a summary of representative porphyrinic MOF composites for biomedical applications. Different porphyrin-based MOFs exhibit different properties and biomedical applications, for example, porphyrinic MOF/PDA@Pt exhibited excellent PTT performance due to PTT characteristics of PDA. Despite the successful synthesis of diverse composite materials, challenges such as intricate preparation procedures, low production efficiency, and high material waste continue to hinder their biomedical development. Moreover, most of the work focused on the Zr-TCPP system, but other metal nodes also exhibited some unique characteristics and they should also be taken seriously. Thus, development of high-efficiency and low-cost porphyrinic MOF composites as biomedical system is vital.

**Table 1:** Examples of porphyrinic MOFs composites used in biomedicine.

Metal nodes	Porphyrin ligand	Other components	Synthesis strategy	Applications	Ref.
Zr	TCPP	Upconvertion nanoparticles	Solvothermal and mixed method	Wound healing and antibacterial therapy	[106]
Zr	TCPP	MXene	Solvothermal and sonication method	Antibacterial therapy	[101]
Zr	TCPP	PDA@FP	Solvothermal and stirred method	Photothermal antibacterial therapy	[107]
Zr	TCPP	Fe SACs	Solvothermal and mixed method	PTT	[108]
Zr	TCPP	PDA@Pt	Solvothermal method	PTT	[98]
Zr	TCPP	Fe_3_O_4_@C	Solvothermal and *in situ* self-assembly method	PDT and PTT	[109]
Zr	TCPP	PCN-224	Hydrothermal method	Sonodynamic therapy	[96]
Mn	TCPP	TCPP	Solvothermal method	Controlled drug delivery and PDT	[110]
Fe	TCPP	BSA/SAs	Solvothermal and sonication method	PDT/PTT	[111]
Cu	TCPP	CSSH-Gel	Solvothermal method and grafting reaction	Synergistic sonodynamic and gas therapy	[112]
Zr	TCPP	PDA	Solvothermal and mixed method	PDT/PTT synergistic antibacterial therapy	[113]
Cu	TCPP	Fe_3_O_4_	Solvothermal method	CDT/PDT	[94]
Zr	TCPP	FAM-P1/P2	Solvothermal method	Fluorescence biosensor	[114]
CuNi	H_3_BTC	CHG	Solvothermal and mixed method	Acetaminophen determination	[102]
Zr	TCPP	Pd nanoparticles	Solvothermal and *in situ* growth method	Oleuropein determination	[115]
Zr	TCPP	CO-COOH	Solvothermal and sonication method	Aptamer-based electrochemical sensor	[116]
In	TCPP	Zn-NO	Solvothermal and calcination method	SDT/GT	[117]

## 3 Biomedical applications of porphyrin-based MOFs

Incorporating porphyrins into MOFs endows these materials with a unique combination of advantageous properties, including outstanding photophysical and electrochemical characteristics, high porosity, modular functionalization capability, and biocompatibility [9,[118], [119], [120]]. These attributes make porphyrin-based MOFs highly attractive for biomedical uses, a field that has garnered increasing research interest. Currently, diverse biomedical applications have been explored, i.e., cancer treatment, antibacterial therapy, wound healing, biosensing platforms, etc. In the following sections, we give a summary of current developments in the biomedical applications of porphyrin-based MOFs.

### 3.1 Cancer treatment

Cancer continues to be a major global health concern since it is one of the most prevalent reasons of death worldwide [[121], [122], [123], [124], [125], [126], [127], [128], [129], [130]]. The three conventional therapeutic modalities such as chemotherapy, surgical intervention, and radiotherapy, have been widely utilized in clinical application for disease control, exhibit inherent limitations. Notably, the non-targeted distribution of chemotherapeutic agents in biological systems, coupled with potential over-treatment or excessive radiation exposure during surgical procedures and radiotherapy, inevitably triggers a spectrum of treatment-related adverse effects [[131], [132], [133]]. A critical limitation of these treatments is their tendency to induce multi-drug resistance and cancer metastasis, ultimately compromising therapeutic efficacy [134]. Given these limitations, there is an urgent need to explore innovative therapeutic paradigms for cancer treatment. A promising approach gaining recent research attention is catalytic therapy, which leverages enzyme-driven chemical reactions exclusively within tumor sites to generate therapeutic agents (e.g., ROS) for targeted cancer elimination while reducing collateral damage to tissue health [[135], [136], [137], [138]]. Among various catalytic nanomaterials, porphyrin-based MOFs have been considered to be the most promising candidates because of their outstanding catalytic activity and minimal cellular toxicity [139]. The porphyrin-based MOFs have shown effectiveness in inducing apoptosis through ROS-mediated mechanisms. For instance, porphyrin-based MOFs with peroxidase (POD)- or oxidase (OXD)-like activity can efficiently convert intratumoral H_2_O_2_ or O_2_ into cytotoxic hydroxyl radicals (·OH) and superoxide radical (O_2_^·-^) through catalytic reactions [97,[140], [141], [142], [143]]. Furthermore, porphyrin-based MOFs exhibit catalase (CAT)-, superoxide dismutase (SOD)-, and glutathione oxidase (GSHOx)-mimicking activities, enabling them to consume H_2_O_2_, O_2_^·-^ and GSH, thereby upsetting the redox homeostasis in the tumor microenvironment (TME) [[144], [145], [146]]. To date, diverse porphyrin-based MOFs with distinct metal nodes and ligand architectures [20,147,148] have been investigated for cancer therapy, aiming to overcome the drawbacks of traditional treatments and consequently increase therapeutic efficacy.

**Fig. 6:**
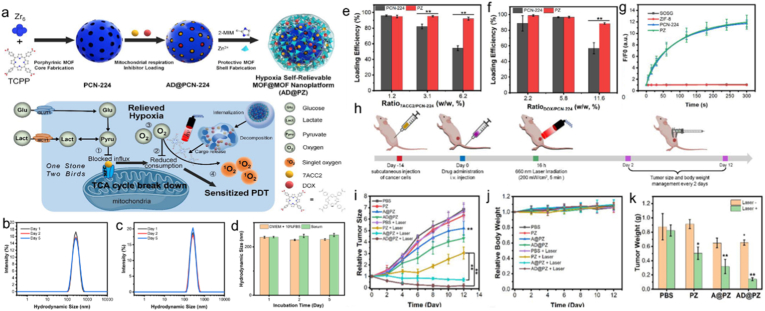
(a) Schematic representation of the manufacturing process for the MOF@MOF nanoplatform with self-relieving hypoxia capability. Size distribution of the MOF@MOF structure incubated in Dulbecco's Modified Eagle's Medium (DMEM) supplied with 10% of fetal bovine serum (FBS) (b) and serum (c) for various durations. (d) Hydrodynamic size changes of the MOF@MOF structure incubated in DMEM supplied with 10% of FBS and serum. Loading efficiency of 7ACC2 (e) and DOX (f) through PCN-224 and PZ. (g) Fluorescence changes of single select oxygen green (SOSG) incubated with ZIF-8, PCN-224, and MOF@MOF (denoted as PZ) under laser irradiation. (h) Diagrammatic representation of the in vivo anti-tumor test protocol. Tumor growth kinetics (i) and mice's body weight control (j) following various treatments. (k) The weight of the tumors that were separated following various treatments. Reproduced from Ref. 150 (For interpretation of the references to colour in this figure legend, the reader is referred to the Web version of this article.)

Commonly, conventional PDT faces efficacy limitations primarily attributed to the hypoxic tumor microenvironment (TME), which significantly hinders therapeutic outcomes [149]. The framework serves as a scaffold to stop porphyrin molecules from aggregating and self-quenching when they are integrated into MOFs. This facilitates efficient energy transfer from porphyrins to oxygen molecules, thereby promoting ROS generation. For example, Tian and co-works [150] proposed a MOF@MOF structure using porphyrin-based MOFs (PCN-224) as core and ZIF-8 as protective layer. This approach significantly improved material stability while alleviating tumor hypoxia, thereby enhancing the efficacy of PDT (Fig. 6a)). The MOF@MOF core-shell structure was remarkably stable thanks to the ZIF-8 shell, which preserved structural integrity under physiological circumstances for 96 h (Fig. 6b–d), which corresponded to its excellent stability. Furthermore, the ZIF-8 shell underwent acid-triggered degradation, enabling the controlled release of the mitochondria-targeted inhibitor (7-amino carboxycoumarin-2, 7ACC2) from the MOF@MOF core-shell structure (Fig. 6e and f). In this system, 7ACC2 suppressed mitochondrial pyruvate uptake while preventing glucose and lactate from powering mitochondrial respiration, thereby alleviating tumor hypoxia. Upon 5-min laser illumination, the 7ACC2-loaded MOF@MOF core-shell nanoplatforms demonstrated a two-fold increase in cellular apoptosis and a 70% reduction in tumor growth in contrast to their cargo-free counterparts (Fig. 6g–k). The development of MOF-based nanomedicines is made possible by this reliable and hypoxia-relieving MOF@MOF platform. Similarly, a near-infrared (NIR) light-adjusted PDT nanoplatform (TPP-UCNPs@MOF-Pt) was reported [151]. TPP-UCNPs@MOF-Pt demonstrates efficient hypoxia alleviation through enzymatic decomposition of intracellular H_2_O_2_ into oxygen, simultaneously elevating ROS levels under NIR light illumination. This cascade reaction significantly enhances the therapeutic outcome of PDT. The work presents a promising approach for developing a porphyrin-based MOFs multifunctional nanoplatform, providing a possible way to overcome PDT's present shortcomings in the treatment of hypoxic malignancies.

In addition to PDT, CDT has been thoroughly investigated for cancer treatment [152,153]. This approach catalyzes endogenous H_2_O_2_ to generate highly active ·OH, offering a promising therapeutic strategy. Nevertheless, these methods yield merely one type of ROS, commonly causing insufficient therapeutic efficacy. In order to improve the therapeutic efficacy, Jiang and co-works [154] described Mn(II) ions chelated porphyrin-based MOFs (Gd-TCPP) nanosheets using a straightforward reflux approach to increase the therapeutic efficacy. These were then coated with pluronic F-127 to produce new sonosensitizers (GMTF). The obtained GMTF nanosheets efficiently generate substantial reactive oxygen species (ROS), while the incorporated Mn(II) ions confer remarkable Fenton-like catalytic activity for enhanced magnetic resonance imaging (MRI)-guided SDT and CDT. In order to overcome the narrow pH range requirement of the Fenton reaction, a Fe_3_O_4_@Cu-TCPP multifunctional nanoplatform was developed [94]. The prepared Fe_3_O_4_@Cu-TCPP exhibited a robust ability to generate ROS via both CDT and PDT pathways, independent of O_2_ availability. This study tackles key challenges including material instability, diminished CDT efficiency under physiological conditions, and the inherent drawbacks of mono-therapeutic approaches, culminating in the development of a multimodal tumor theranostic agent.

PTT, which employs NIR light and various photothermal agents, is widely used for tumor ablation by converting light energy into heat. This approach complements the CDT/PDT strategies discussed earlier, offering a synergistic thermal-based treatment modality [155,156]. Besides excellent catalytic therapies, porphyrin-based MOFs also display good photothermal effects for efficient PTT because of the inherent features of porphyrin structure [157]. Recently, Zhou and co-workers [108] synthesized a porphyrin-based MOF featuring abundant Fe(III) centers (P-MOF). Owing to its high density of these single-atom Fe(III) active sites, through photoacoustic imaging (PAI)-guided PTT/PDT, the P-MOF demonstrated exceptional effectiveness in modifying the hypoxia tumor microenvironment of Hela cell tumors in mice. Computational analyses revealed that the material's narrow bandgap energy (1.31 eV) enabled efficient absorption of near-infrared (NIR) photons, thereby inducing nonradiative transitions that converted incident light into heat to facilitate PTT. Similarly, a smart Zr/Co porphyrin-based MOF nanotheranostic system demonstrated exceptional photothermal conversion capability while simultaneously achieving abundant singlet oxygen (^1^O_2_) production, thereby enabling potentiated cancer therapy through fluorescence imaging (FLI)/photothermal imaging (PTI)-guided PTT [158]. This study offers a fresh perspective on creating more effective and safer nano-based multimodal therapies, aiding their future clinical application.

**Fig. 7:**
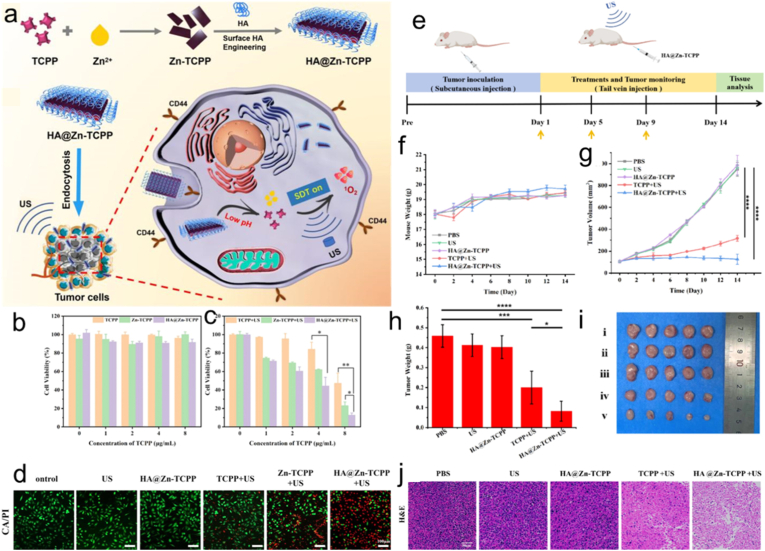
(a) Synthesis of HA@Zn-TCPP for activating SDT in vivo. Cell viability of 4T1 cells treated with free TCPP, Zn-TCPP, and HA@Zn-TCPP with (b) or without (c) ultrasound irradiation. (d) Calcein-AM/propidium iodide staining images of 4T1 cells in various groups. (e) Description of the SDT process in vivo. Changes in (f) body weight and (g) tumor volume of mice with various treatments in 14 days. (h) Tumor weight of mice with various groups after 14 days. (i) Tumor images after 14 days. (j) H&E staining images of tumors in different treatments after 14 days. Reproduced from Ref. [162].

SDT leverages low-intensity ultrasound and sonosensitizers to enable deep tumor treatment, offering a promising clinical approach [3,159,160]. Current sonosensitizers are hindered by limited tumor accumulation, inefficient ROS generation under ultrasound, and poor biodegradability, all of which constrain therapeutic efficacy. Furthermore, the complex and immunosuppressive TME presents a major barrier, making it difficult for SDT alone to achieve complete tumor eradication [161]. Chen and co-workers [162] introduced a new-type zinc(II)-porphyrin-based nanotheranostic platform (HA@Zn-TCPP) through a straightforward thermal reaction (Fig. 7a). Tumor cells were able to efficiently internalize HA@Zn-TCPP thanks to HA-mediated targeting, which resulted in its preferential concentration on tumor tissues that overexpress CD44. Under acidic conditions, the nanotheranostic platform underwent rapid decomposition, liberating free TCPP to activate SDT (Fig. 7b–d). Consequently, the HA@Zn-TCPP nanotheranostic system demonstrated outstanding tumor-suppressive efficacy, achieving an 82.1% inhibition rate under ultrasound irradiation (Fig. 7e–j). This discovery establishes a critical methodology for engineering next-generation sonosensitizers with enhanced SDT efficacy. Furthermore, a porphyrin-based MOF was also incorporated into a double-network hyaluronic acid (HA) hydrogel to form a tumor-responsive nanocomposite, thereby enhancing sonodynamic therapy (SDT) efficacy [112]. It also exhibited excellent injectability and biocompatibility. Thus, porphyrin-based MOFs exhibit great potential for SDT.

To augment SDT efficacy, gas therapy (GT) has emerged as a promising strategy, leveraging its selective cytotoxicity against cancer cells while preserving normal tissue integrity [163,164]. NO stands out among the key gasotransmitters (NO, CO, H_2_S) in gas therapy research, owing to its complex involvement in cancer processes and considerable therapeutic promise [123]. Low concentrations of NO promote tumor growth, while higher levels (>1 μM) trigger tumor cell apoptosis via mitochondrial and DNA oxidation or nitrosation. Recent research has extensively explored both endogenous and exogenous stimuli for the controlled release of NO from various donors and precursors in porphyrin-based MOFs [165,166]. For instance, Chen and co-workers [117] reported 2D NO-functionalized porphyrin-based MOFs nanosheets, enabling ultrasound-triggered synergistic GT and SDT for cancer treatment. The NO-functionalized MOF (In-TCPP@Zn-NO) was produced through sequentially coordinating and adsorbing NO gas after Zn^2+^ were first chelated to the porphyrin core of the MOF (In-TCPP). Under ultrasound irradiation, the treatment not only promoted robust ROS generation but also triggered rapid and efficient NO release from the In-TCPP@Zn-NO nanosheets. The prepared In-TCPP@Zn-NO composite exhibited ultrasound-triggered NO release behavior, with significantly improved kinetics and unique features of temporal adjustment. Thus, ultrasound activated the In-TCPP@Zn-NO nanosheets to simultaneously produce ROS and released NO, thereby synergistically enhancing the efficacy of SDT and achieving complete eradication of tumors. In order to achieve extremely effective cancer treatment, this study provides a novel nanoplatform design technique with combined dual-functional sites that enable synergistic GT/SDT while successfully resolving the temporal and environmental restrictions of GT.

### 3.2 Antibacterial therapy

Currently, bacterial infections rank among the leading global health challenges, impacting millions annually [[167], [168], [169], [170], [171]]. Conventional treatment approaches depend excessively on antibiotics, accelerating the rise of multi-drug resistance (MDR) and undermining therapeutic efficacy [172,173]. To address these limitations, research into antibiotic-free alternatives has garnered significant attention [174]. Porphyrin-based MOFs with OXD- or POD-like activity can generate ROS from H_2_O_2_ and O_2_, which disrupt bacterial cell membranes, DNA, and proteins to achieve bactericidal activity and is considered to be one of the most effective strategies [19]. This ROS-driven sterilization strategy effectively circumvents bacterial resistance and minimizes biological toxicity, demonstrating broad-spectrum antibacterial efficacy distinct from conventional antibiotics.

**Fig. 8:**
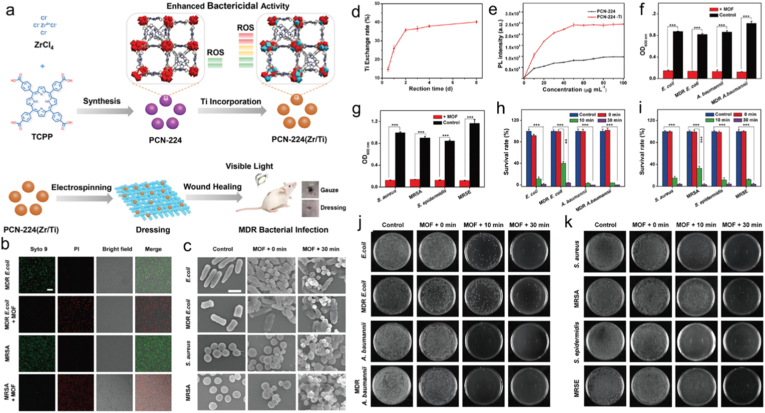
(a) Production of PCN-224 (Zr/Ti) to treat bacterial infections. (b) Magnified pictures of MRSA and MDR *E. coli* treated with or without PCN-224 (Zr/Ti). (c) SEM pictures of variously treated bacteria. (d) Ti exchange rate using the titanium inclusion approach with varying reaction durations. (e) PL spectra of DCFH as a sensor to identify the production of ROS from PCN-224 (black) and PCN-224 (Zr/Ti) (red) at different concentrations when exposed to visible light for 3 min (f-g) The OD 600 nm values at 24 h of PCN-224 (Zr/Ti)-treated or untreated Gram-positive and Gram-negative bacteria exposed to visible light for 30 min. (h-i) Gram-positive and Gram-negative bacterial survival rates in incubation. (j-k) Images of Gram-positive and Gram-negative bacteria that were exposed to PCN-224 (Zr/Ti) for 0, 10, and 30 min, respectively. Reproduced from Ref. 177 (For interpretation of the references to colour in this figure legend, the reader is referred to the Web version of this article.)

Despite high photosensitizer (PS) efficiency, biofilm eradication demands elevated light/PS concentrations due to restricted penetration and diffusion within biofilms. Moreover, the hypoxic microenvironment and rapid oxygen depletion during PDT significantly constrain antibacterial therapy efficacy [175]. In order to overcome these issues, a porphyrin-based MOF with enhanced fouling penetration, self-oxygenation, and photodynamic performance was developed for bacterial biofilm eradication [176]. The system comprises porphyrin-based MOF dots encapsulated in human serum albumin-coated MnO_2_. In biofilms, pH/H_2_O_2_-triggered MnO_2_ degradation released ultrasmall porphyrin-based MOF dots with positive charge and O_2_ *in situ*, effectively alleviating hypoxia. The released porphyrin-based MOF dots, exhibited high ROS yield, penetrate biofilms, bind to bacterial surfaces, and enable biofilm ablation. Bimetallic PCN-224(Zr/Ti) was fabricated through a facile cation exchange strategy (Fig. 8a), which demonstrated significantly improved capability for ROS generation, which underpined its efficient antibacterial performance [177]. The prepared PCN-224(Zr/Ti) were incorporated into lactic-co-glycolic acid microfibers to create a wound care material demonstrated excellent biocompatibility and negligible toxicity (Fig. 8b and c). This innovative dressing proved effective for in vivo PDT-assisted healing of persistent wounds infested with multidrug-resistant (MDR) bacteria (Fig. 8d–k). This study employs an antibiotic-free approach by utilizing the inherent properties of porphyrin-based MOFs, offering a cost-effective and facile solution that fully exploits their potential as potent nonantibiotic agents in PDT.

PDT holds great promise for treating bacteria-infected osteomyelitis owing to its deep tissue penetration and independence from antibiotic use [[178], [179], [180]]. Nevertheless, the majority of photosensitizers suffer from inefficient electron transfer, leading to inadequate generation of ROS [181]. By anchoring Pt single-atom catalysts (SAs) onto 2D Al-TCPP MOF nanosheets, the dispersion and stability of Pt atoms were enhanced [182]. This integration also leveraged the porphyrinic MOF's crystalline porous structure to synergistically interact with Pt SAs, thereby maximizing the capacity to trap light. This distinctive configuration strengthened the bridging linkage between Pt single-atom sites and the porphyrin framework, enabling rapid charge migration and separation upon light exposure, thereby augmenting ROS generation. Studies conducted both *in vitro* and in vivo verified that Pt/Al-TCPP nanosheets quickly eliminated germs at low levels when exposed to laser radiation, leveraging their optimized light-trapping and charge separation capabilities. The Pt SA-decorated 2D MOF nanosheets hold significant promise for antibacterial applications, providing innovative strategies to treat bacterial infections through their enhanced light absorption and efficient charge transfer properties.

Compared to bulk MOFs, the fabrication of 2D porphyrin-based MOFs enhances the accessibility of active sites by maximizing the surface-area-to-volume ratio, thereby improving light-driven charge transfer and ROS generation efficiency [13]. Tan and co-workers [183] developed a surfactant-free, solvent-mediated synthesis approach to prepare 2D porphyrin-based MOF (In-TCPP) nanosheets, which exhibited improved photodynamic antibacterial efficiency. The shape of 2D In-TCPP nanosheets was easily to be precisely controlled through adjusting the water-to-DMF ratio with pyridine assistance. As a result, compared to bulk In-TCPP, the morphologically optimized 2D nanosheets demonstrated superior ^1^O_2_ generation under 660 nm laser exposure, leveraging their expanded active site accessibility to enhance photodynamic antibacterial therapy. A porphyrin-based MOF micromotor was also used for effective photocatalytic antibacterial applications [184]. Metal doping enhanced ROS generation and induced a more positive surface charge upon light exposure, both of which were critical for short-range antibacterial efficacy.

### 3.3 Wound healing

Bacterial wound infections pose a critical threat to public health, and photocatalytic technology offers a promising solution for achieving effective wound healing [[185], [186], [187], [188]]. Porphyrin-based MOFs alleviate long-term oxidative stress by mimicking antioxidant enzyme activities to scavenge over-accumulated ROS and protect cells from oxidative damage. Furthermore, they intervene in the local immune microenvironment by reducing ROS-induced pro-inflammatory cytokine secretion and modulating macrophage polarization from the pro-inflammatory M1 phenotype toward the pro-regenerative M2 phenotype, thereby creating a favorable milieu for tissue repair [189,190].

**Fig. 9:**
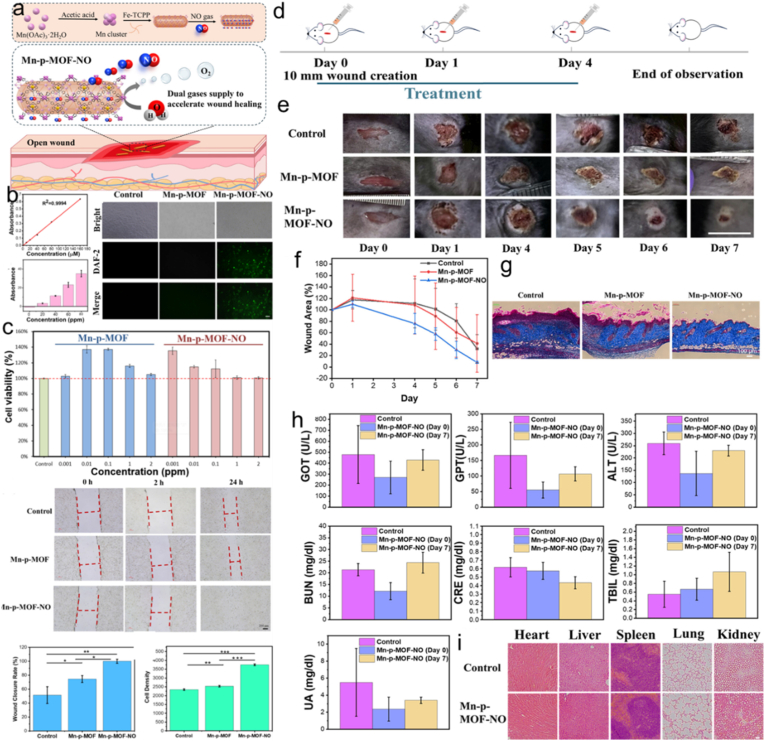
(a) Combination gas therapy utilizing Mn-p-MOF-NO for enhanced wound healing. (b) Assessment of the production of NO by Mn-p-MOF-NO. (c) *In vitro* investigations of Mn-p-MOF-NO. (d) The timetable of the animal investigation's wound formation, therapy, and assessment. (e) Images of wounds treated and untreated with Mn-p-MOF and Mn-p-MOF-NO, respectively. (f) Examination of wound dimensions in mice treated with MOF and those not. (g) Pictures of skin cells stained with Masson's trichrome after seven days. Mice treated and untreated with Mn-p-MOF-NO were subjected to blood biochemistry (h) and histochemistry tests (i). Reproduced from Ref. [195].

Porphyrin, as a key ligand in MOF frameworks, harnesses its intrinsic photosensitivity to generate ROS upon light exposure, offering a promising approach for wound healing through photodynamic therapy [191,192]. Medical gases such as oxygen and NO have garnered considerable clinical attention for wound healing applications, owing to their ability to promote cellular proliferation, stimulate angiogenesis, and facilitate collagen deposition [193,194]. Kan and co-workers [195] demonstrated a viable strategy employing a porphyrin-based MOF (Mn-p-MOF), a novel material with remarkable promise for dual-gas-enhanced wound treatment (Fig. 9a). Catalytically active Mn clusters in precisely designed MOF nanorods promoted spontaneously H_2_O dissolution and O_2_ evolution upon aqueous contact. Fe-coordinated porphyrins, which were included into the MOF, showed a high affinity for NO gas and served as receptors for Mn clusters, enabling effective NO delivery (Fig. 9b). Upon incorporation into fibroblast cell cultures, the MOF demonstrated enhanced O_2_ and NO supply, leading to boosted cell transfer and proliferation *in vitro* (Fig. 9c) and in vivo (Fig. 9d–i). The material's promise in H_2_O-powered dual-gas treatment for wound healing applications is strongly supported by this discover.

The broad energy band gap and inefficient electron transfer of emerging porphyrin-based MOF sonosensitizers significantly limit the therapeutic efficacy of SDT [96,196]. Dong and co-workers [197] developed an engineered porphyrin-based MOF composite by integrating multivariate MOFs with *in-situ* surface loading of Ag nanoparticles (NPs), significantly improving the SDT efficiency against bacterial infections in wound healing. The band gap was lowered from 3.45 to 2.11 eV by integrating TCPP into Zr nodes and then loading Ag NPs. This significant narrowing enhanced electron-hole pair generation under ultrasound illumination. Simultaneously, the introduction of Ag NPs enhanced electron migration kinetics. As a result, the Ag@UT system demonstrated robust antibacterial activity *in vitro* and boosted wound treatment in vivo under ultrasound stimulation. This study provides insightful information about MOF optimization and strategic thinking, achieved through the synergistic integration of tailored ligands and surface functionalization. This approach enables advanced SDT performance, paving the path for effective antibacterial solutions in wound healing applications.

The efficacy of PDT in treating diabetic infectious wounds is promising, but it faces challenges from insufficient local O_2_/H_2_O_2_ generation and persistent inflammation [198]. The multidimensional regulation of diabetic infected wounds involves a complex interplay between host pathophysiology, microbial virulence, and polymicrobial dynamics. Hyperglycemia impairs host immune responses while simultaneously enhancing bacterial virulence factors, such as *S. aureus* glucose transporters and proteases, leading to more severe infections. The wound microenvironment promotes polymicrobial biofilm formation, which protects pathogens like *P. aeruginosa* and *S. aureus* from antibiotics and host immunity. Additionally, metabolic alterations in the wound shape microbial communities, often favoring anaerobes associated with poor healing. These interconnected factors collectively determine infection progression and outcomes in diabetic wounds [199,200]. Recently, Li and co-workers [201] designed a nanospray (PIQS) with Ir NPs-modified porphyrin-based MOF, capable of responding to both infection and inflammation. The findings revealed that PIQS displayed potent anti-infective effects versus *E. coli* and *S. aureus* when exposed to 660 nm laser light, leveraging improved PDT/CDT. Its diverse enzyme-mimetic properties, which include GO_x_, POD, SOD, and CAT activities, account for its effectiveness. By neutralizing ROS, PIQS's Ir NPs lower oxidative stress and cause macrophages to switch from the M1 pro-inflammatory to the M2 anti-inflammatory state. The reported PIQS promoted immune balance, epithelial repair, and skin regeneration, showcasing its nanospray as an innovative solution for complex diabetic wounds with infections. Shi and co-workers [202] developed a Cu^2+^-doped porphyrin MOF (MOF@Cu^2+^) for improved photodynamic treatment of biofilm infections by maximizing GSH depletion. The Cu^2+^ released from the MOF@Cu^2+^ framework serves two key roles: oxidizing GSH in the biofilm and consuming GSH released by bacteria subjected to ROS attack. As a result, the antioxidant capacity of both the biofilm and bacterial cells is severely impaired, while ROS levels are significantly increased. Thus, the porphyrin-based MOF nanosystem provides reliable and secure simultaneous PDT therapeutics for wound healing.

### 3.4 Biosensors

Recently, there has been a growing interest in the development of very sensitive biosensors. [[203], [204], [205], [206]]. Thanks to their distinctive physicochemical characteristics, functional nanomaterials enable signal amplification, driving dramatic progress in biosensing applications [207]. Porphyrin-based MOFs have been extensively documented in biosensors with high efficiency, high selectivity, and extended target range because of their excellent photophysical features e.g. fluorescent nature, high surface area and controllable porosity [208,209]. In the following section, an overview of porphyrin-based MOFs for biosensing uses will be presented.

Accurate and extremely sensitive nucleic acid biomarker detection is essential for preliminary cancer screening, efficient disease detection, and effective clinical therapy [210]. To achieve sensitive and efficient nucleic acid biomarker detection, Gao and co-workers [114] developed a switchable fluorescence biosensor. The sensor used FAM-modified ssDNA probes (FAM-P1/P2) and Zr-MOF, with the latter acting as a fluorescence quencher. The adsorption of FAM-P1/P2 onto Zr-MOF, driven by intermolecular forces, triggered fluorescence quenching through fluorescence resonance energy transfer (FRET) and photo-induced electron transfer (PET) mechanisms. Consequently, the fluorescence mode went "off" for the system. The robust double-stranded DNA (dsDNA) structures separated from the Zr-MOF surface when the fluorescence probes specifically bound to their targets. This release triggered fluorescence recovery, transitioning the system into an "on" state. Owing to ZrMOF's stronger affinity for ssDNA than dsDNA, the fluorescence signal transitioned from "off" to "on," enabling rapid and ultrasensitive detection of ssDNA (T1) and microRNA-21 (miR-21) within 30 min, surpassing that of most of MOF-based platforms reported in recent years. Thus, this biosensor holds promise as a potential candidate for detecting DNA and miRNA biomarkers.

**Fig. 10:**
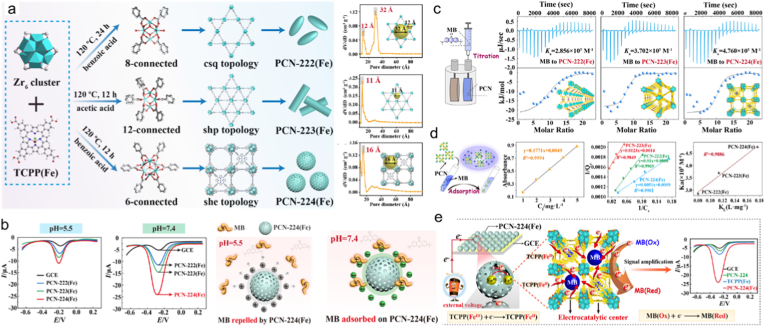
(a) Synthetic route and pore-size distribution of PCN-222(Fe), PCN-223(Fe), and PCN-224(Fe). (b) Square wave voltammetry measurements of three PCN-modified GCEs at HAc-NaAc (0.1 M, pH 5.5) and PBS (0.1 M, pH 7.4) containing 25 μM of MB and electrostatic repulsion or adsorption between PCN- 224(Fe) and methylene blue (MB). (c) Schematic of the ITC titration between MB and PCNs. (d) Schematic of MB adsorption in PCNs. (e) Electron transfer process occurs from PCN-224(Fe) toward MB. Reproduced from Ref. 213 (For interpretation of the references to colour in this figure legend, the reader is referred to the Web version of this article.)

Modulating the pore structure of porphyrin-based MOFs is essential for customizing their catalytic behavior and advancing biosensor applications [211]. However, establishing precise control over the interplay between porphyrin-based MOFs' pore architecture and catalytic functionality continues to pose significant challenges [212]. Recently, Chen et al. [213] reported how pore size and topology govern electrochemical catalytic performance, employing three Fe-based porphyrinic MOFs with various connecting numbers and topologies, e.g., PCN-222(Fe), PCN-223(Fe), and PCN-224(Fe) (Fig. 10a). Among these series, PCN-224(Fe) exhibited superior electrochemical activity, a performance attributable to the close alignment between its pore dimensions and the spatial distribution of active site (Fig. 10b). Using isothermal titration calorimetry (ITC), a strong PCN-224(Fe)-MB interaction through the highest association constants and lowest entropy-driven contributions were found (Fig. 10c and d). Density functional theory (DFT) computations revealed how d-π coupling and π-π stacking work in concert and outlined the electron migration channel in PCN-224(Fe), offering atomic-level understanding into the active catalytic process (Fig. 10e). This study elucidates porphyrin-based MOFs’ pore structure-property correlations in electrochemical (EC) biosensing through integrated thermodynamic and computational perspectives.

Porphyrin-based MOFs, constructed from customized porphyrin-based ligands, exhibit strong potential in photoelectrochemical (PEC) biosensing because of their exceptional biocompatibility and versatile biophotochemical functions [214]. Zhang and co-workers [215] demonstrated that 3D interpenetrated porphyrin-based MOFs built from 5,10,15,20-tetra(4-pyridyl)porphyrin (TPyP) showed improved electron delocalization and migration through rich π-π* interactions of porphyrin units. Based on this result, a Bi_2_S_3_@Cu-TPyP heterojunction was successfully utilized as a PEC sensor by Zhang's group, enabling ultrasensitive gentamicin (GEN) detection in complex food matrices [216]. The synergistic combination of Cu-TPyP's electron migration enhancement, photoabsorption improvement, high surface area, and antibody affinity with Bi_2_S_3_'s narrow bandgap created an optimal interface contact. Optimized charge carrier separation and boosted conversion efficiency synergistically contribute to the photocurrent amplification observed in the Bi_2_S_3_@Cu-TPyP heterojunction. By combining synergistic signal amplification with maintained specificity, this work establishes a food-compatible PEC biosensor for antibiotic detection.

### 3.5 Others

Apart from the aforementioned biomedical uses, porphyrin-based MOFs have also been widely used in other applications such as nerve repair, bone regeneration, targeted control of plant diseases, etc. In the following section, we provide an overview of recent advancements in the other biomedical applications using porphyrin-based MOFs.

Neurological disorders are increasingly contributing to global mortality and disability. Repairing nervous system injuries remains particularly challenging, mainly because of the limited regenerative capacity of neurons and the complexity of the neural microenvironment [217]. Recently, Dou and co-workers [218] reported that systematic defect engineering of MOF-525 modulated the accessibility of Zr clusters and the molecular sieving properties of its internal channels. This approach enabled precise fluorescent detection of phosphoryl fluoride nerve agents, based on their distinct chemical reactivity and molecular dimensions. This system demonstrated high sensitivity and rapid response to target nerve agents, while maintaining robustness against interference from acidic, humid conditions and common fluorescent species. This work opens a new avenue for detecting trace nerve agent vapor. A single intraocular injection of Zr-MOF also accelerated photoreceptor regeneration and restored visual function in the injured retina by promoting cell proliferation, reducing inflammatory responses, and enhancing antioxidase expression in age-related macular degeneration (AMD) therapy [219]. This work offers strategic guidance for the use of porphyrin-based MOFs in nerve repair.

**Fig. 11:**
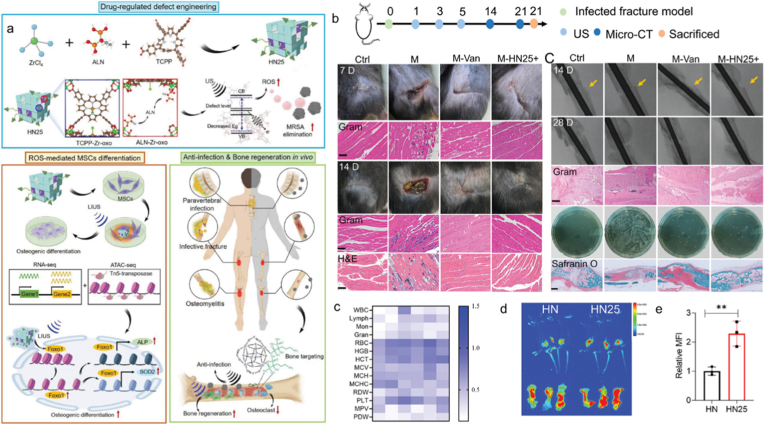
(a) Schematic diagram illustrating the HN25 for targeted bone tissue healing via sonically modulated chromatin accessibility. (b) In vivo treatment of diseased bone fractures using bone-targeting HN25. (c) Blood analysis after 28 days. (d) In vivo and ex vivo fluorescence imaging of the fracture site 30 min post HN or HN25 injection. (e) Quantitative fluorescence intensity of mouse bone tissues. Reproduced from Ref. [222].

For infectious bone diseases, it is essential to both eliminate bacterial infections and simultaneously promote osteogenic differentiation [220]. Elevated levels of ROS can induce cellular damage, whereas at low concentrations, ROS act as signaling molecules that regulate cell fate [221]. An alendronate (ALN)-mediated defective porphyrin-based MOF sonosensitizer was recently developed [222] (Fig. 11a). This material enabled effective clearance of methicillin-resistant *Staphylococcus aureus* (MRSA) infections and promotes osteogenic differentiation when exposed to different ultrasound treatmen. As a result, the produced ALN-mediated porphyrin-based MOF (HN25) with an appropriate vacancy demonstrated excellent bone-targeting and SDT efficiency. HN25 achieved targeted and effective repair of diverse infected bone tissues (Fig. 11b–e). This was accomplished through rapid MRSA clearance, suppression of osteoclast activity, and promotion of bone regeneration. This work presents a promising therapeutic strategy for the regeneration of infected tissues. Moreover, PCN/β-Ca_2_SiO_4_ composite scaffolds also enhanced cell proliferation and upregulated osteogenesis-related gene expression [223]. Excellent biocompatibility was also exhibited in the composite scaffolds.

Nanotechnology offers groundbreaking solutions to enduring problems in crop protection [224]. By releasing their payload in accordance with specific cues (i.e., pH, enzymes, and light), stimuli-responsive nanopesticides enhance efficacy through targeted delivery while reducing off-target loss [225]. Porphyrin-based MOFs is a promising intelligent pesticide delivery system for crop protection because of their outstanding chemical stability, suitable electronic structure, and photoresponsivity nature [226]. Uniform PCN-222 nanoparticles (⁓140 nm) were first synthesized via a trifluoroacetic acid-mediated method. Subsequently, Mg^2+^ doping combined with polydopamine-assisted encapsulation of prochloraz (Pro) yielded functional PCN-222(Mg)@Pro@PDA architectures, which exhibited a high loading capacity (21.08%) and a mesoporous structure. This synergistic system functions via a complementary mechanism: acidic microenvironments increase PDA permeability to trigger fungicide release, while visible light enhances ROS generation within the Mg-porphyrinic network, collectively improving antifungal efficacy [227]. This work integrates modulator-controlled MOF precision with dual environmental responsiveness, providing a rational strategy for intelligent, eco-compatible pesticide delivery in sustainable agriculture.

In summary, porphyrin-based MOFs have been used extensively in biomedical domains, i.e., cancer therapy, antibacterial treatment, wound repair, and biosensing (Table 2). To ensure precise diagnosis and effective treatment, rational design of these porphyrin-based MOFs requires careful control over their morphology, composition, surface engineering, and functionalization with targeting groups or bioactive components for safe and efficient performance. Additionally, optimizing their size, shape, chemical composition, and surface features is crucial to enhance their therapeutic efficacy and biosensing capabilities, thereby meeting the demands of personalized medicine and diagnostics.

**Table 2:** An overview of typical porphyrin-based MOFs for possible applications in medicine.

Materials	Metal nodes	Porphyrin ligand	Other components	Catalytic mechanisms	Biomedical application	Ref.
MOF@MOF	Zr	TCPP	ZIF-8	OXD mimicking	Cancer therapy (PDT)	[150]
TPP-UCNPs @MOF-Pt	Zr	TCPP	TPP-UCNPs-Pt	POD mimicking	Cancer therapy (PDT combined with mitochondrial targeting and NIR activation)	[151]
GMTF	Gd	TCPP	Pluronic F-127	CAT mimicking	Cancer therapy (MRI-guided SDT/CDT)	[154]
Fe_3_O_4_@ Cu-TCPP	Cu	TCPP	Fe_3_O_4_	OXD mimicking	Cancer therapy (PDT/CDT)	[94]
P-MOF	Fe(III)	TCPP	-	OXD mimicking	Cancer therapy (PAI-guided PTT/PDT)	[108]
Zr-TCPP(Co)/ICG/PEG/Apt-M	Zr/Co	TCPP	ICG/PEG/Apt-M	POD mimicking	Cancer therapy (FLI/PTI-guided PTT)	[158]
HA@Zn-TCPP	Zn	TCPP	HA	POD mimicking	Cancer therapy (SDT)	[162]
CSSH-Gel	Cu	TCPP	HA hydrogel	CAT mimicking	Cancer therapy (SDT/GT)	[112]
In-TCPP@ Zn-NO	In	TCPP	Zn-NO	POD mimicking	Cancer therapy (SDT/GT)	[117]
PCN-224(Zr/Ti)	Zr/Ti	TCPP	-	POD mimicking	Antibacterial therapy (PDT)	[177]
Pt/Al-TCPP	Al	TCPP	Pt single-atoms	POD mimicking	Antibacterial therapy (PDT/PTT)	[182]
In-TCPP nanosheets	In	TCPP	-	OXD mimicking	Antibacterial therapy (PDT)	[183]
PCN-224 MOFtors	Fe or Cu	TCPP	-	CAT mimicking	Antibacterial therapy (PTT)	[184]
Mn-p-MOF	Fe	TCPP	-	CAT mimicking	Wound healing (GT)	[195]
Ag@UT	Zr	TCPP	Ag	POD mimicking	Wound healing (SDT)	[197]
PIQS	Zr	TCPP	Ir nanoparticles	SOD mimicking	Wound healing (CDT/PDT)	[201]
MOF@Cu^2+^	Zr	TCPP	Cu^2+^	GO_x_, POD, SOD, and CAT mimicking	Wound healing (PDT)	[202]
ZrMOF- FAM-P1/P2	Zr	TCPP	FAM-P1/P2	-	Biosensors (detecting DNA and miRNA biomarkers)	[114]
MB@PCN-224(Fe)	Fe	TCPP	MB	-	Biosensors (EC)	[213]
ZnTPyP-1	Zn	TPyP	-	-	Biosensors (PEC)	[215]
Bi_2_S_3_@Cu-TPyP	Cu	TPyP	Bi_2_S_3_	-	Biosensors (PEC)	[216]
MOF-525	Zr	TCPP	-	-	Nerve repair (fluorescent)	[218]
Zr-MOF	Zr	TCPP	-	-	Nerve repair (AMD therapy)	[219]
HN25	Zr	TCPP	ALN	POD mimicking	Bone regeneration (SDT)	[222]
PCN/β-Ca_2_SiO_4_	Zr	TCPP	β-Ca_2_SiO_4_	-	Bone regeneration (osteogenesis)	[223]
PCN-222(Mg)@ Pro@PDA	Mg	TCPP	Pro@PDA	-	Crop protection (intelligent pesticide delivery system)	[227]

## 4 Biosafety and toxicity evaluations

The biosafety and toxicity of porphyrin-based MOFs are key determinants of their potential for clinical application. In vivo toxicological and pharmacokinetic studies clarify their absorption, distribution, metabolism, excretion, and clearance, thereby enabling a comprehensive evaluation of their biosafety and toxicological characteristics. Given that porphyrin-based MOFs are assembled from metal centers and organic ligands, selecting building components with low or no toxicity is essential to enhance biocompatibility and minimize potential toxicity [8]. Porphyrins, serving as biocompatible building motifs, contribute positively to the biosafety of MOFs. Nevertheless, the toxicity of these frameworks is still largely determined by their metal nodes [228,229]. To construct biocompatible porphyrin-based MOFs for biomedical use, metal ions with low intrinsic toxicity such as Zr, Fe, Zn, Ca, and Mg, etc, which can serve as suitable structural nodes [230]. When synthesizing composite porphyrinic MOFs, assessing the possible biological effects of any integrated functional components is also crucial. Beyond composition, key physicochemical features including particle size, shape, surface characteristics, and dose-dependent behaviors, which significantly influence the toxicological profile of these materials [231,232]. The aforementioned factors significantly impact porphyrin-based MOFs' intake, distribution, metabolism, elimination, and decomposition, which in turn shapes their in vivo toxicological profile. However, while the multifunctionality of these materials has been extensively studied in biomedical research, these key toxicological aspects remain insufficiently explored.

**Fig. 12:**
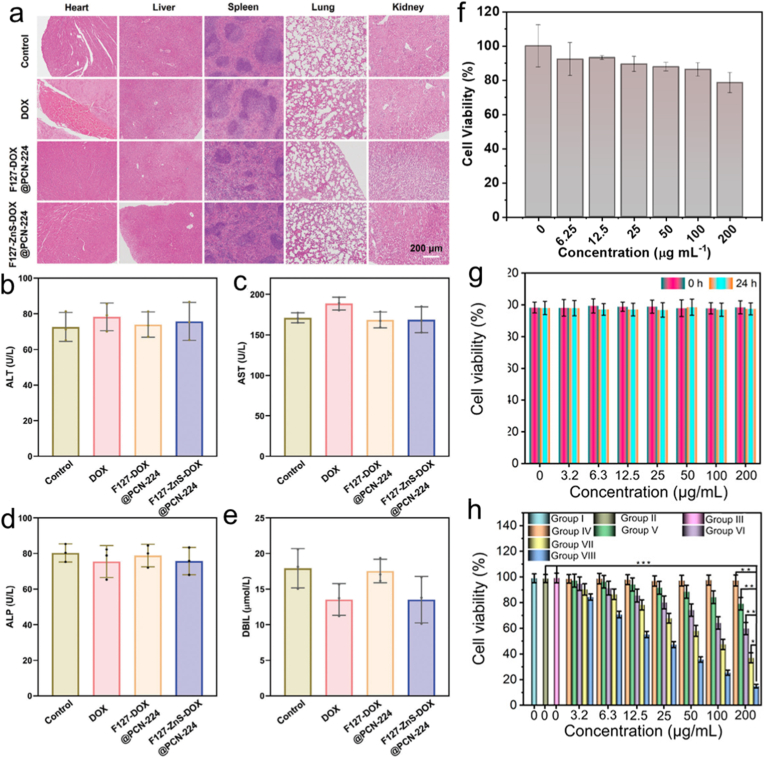
(a) After 12 days of various therapies, the primary organs of tumor-bearing mice were stained with H&E. (b-e) Serum biochemical examinations of mice in different groups of therapy to evaluate liver and kidney function. Reproduced from Ref. [234] (f) Survivability of 3T3 cells exposed to varying FMP doses. Reproduced from Ref. [235]. (g) L929 fibroblast cell viability after 12 and 24 h of UPFB incubation. (h) The vitality of cells under various conditions. Reproduced from Ref. [236].

In order to study the biosafety and toxicity of porphyrin-based MOFs [23,233], many researches have been carried out. For example, a self-propelled nanorobot powered by light and constructed from a porphyrin-based MOF was presented for the intelligent co-delivery of chemotherapeutic agents and photosensitizers to achieve deep tumor penetration [234]. Tumor tissues and primary organs were removed from nude mice for hematoxylin and eosin (H&E) staining, the results confirmed that the prepared nanocomposites exhibited minimal damage to vital organs (Fig. 12a). Further in vivo safety assessment of the prepared nanocomposites involved serum biochemical tests for hepatic and renal function. Neither significant changes in these indices versus the control, nor values outside the normal range were observed, demonstrating that the treatments did not impair liver or kidney function (Fig. 12b–e). In addition, a novel Fe(TCPP)-MOF was also designed for enhancing ROS production for ultrasound-triggered pyroptosis [235]. In subsequent cytotoxicity assessments, the effects of NH_2_-PEGCOOH-coated Fe(TCPP)-MOF (FMP) on murine breast cancer cell line (4T1) cells and normal mouse embryonic fibroblasts (3T3) were assessed via the standard MTT assay. A robust viability of 78% was observed in 3T3 cells even at 200 μg/mL, further confirming the good biocompatibility of FMP (Fig. 12f). Similarly, long-term in vivo data exhibited that even at concentrations up to 200 μg/mL, L929 cells maintained high biocompatibility following 24-h incubation with an upconverted porphyrin-based MOFs nanocomposite (UPFB) (Fig. 12g and h) [236]. The results above demonstrate that the porphyrin-based MOFs exhibit low systemic toxicity and favorable biocompatibility, and are efficiently eliminated via renal clearance post-treatment.

In summary, the study of biosafety and toxicity for porphyrin-based MOFs remains at an early stage. Preliminary *in vitro* and in vivo assessments of toxicity and acute effects in mice have constituted the majority of existing studies. While current evidence indicates favorable biosafety and minimal toxicity at tested doses, key aspects e.g., biodistribution, tolerance thresholds, degradation pathways, and clearance mechanisms, which remain insufficiently explored. Furthermore, their retention, excretion patterns, potential long-term toxicity, and overall impact in vivo must be thoroughly characterized to support future clinical translation. Therefore, more systematic and in-depth investigations into the safety profile of porphyrin-based MOFs are urgently needed.

## 5 Conclusions and outlook

In this review, we methodically examine the design principles and biosafety evaluations for porphyrin-based MOFs. Additionally, we highlight latest developments in their biomedical uses such as cancer therapy, antibacterial treatment, wound healing, and biosensor. Their exceptional structural and functional properties, good biosafety and low toxicity and intrinsic biodegradability ensure them with considerable potential in biomedical applications. Although porphyrin-based MOFs are garnering increasing interest, the field remains nascent and demands further investigation. Considering both the opportunities and challenges in advancing these materials for biomedical use, we offer the following perspectives.

(1) Developing cost-effective and high-performance porphyrin-based MOFs is vital for actual clinical application. Moreover, the practical application is also hindered by complex ligand synthesis, demanding material processing, and suboptimal optoelectronic properties. Addressing these challenges demands significant research and development, which is both technically demanding and costly.
(2) The excitation of porphyrin-based MOFs is currently mostly limited to a wavelength of around 660 nm, and in clinical applications, it needs to be extended to longer wavelengths to achieve deeper tissue penetration performance.
(3) Currently, significant gaps persist in our understanding of the in vivo safety, metabolic paths, mechanisms of action, and long-lasting toxicology of porphyrin-based MOFs. Moving forward, investigations should prioritize the development of materials with enhanced biosafety, minimized toxicity, improved targeting precision, and efficient in vivo delivery.
(4) In porphyrin-based MOFs, the photoelectrochemical performance is largely governed by the interaction between the porphyrin linkers and the metal nodes. Within these structures, the porphyrin ligands generally serve as electron donors, whereas the metal nodes commonly act as electron acceptors. Consequently, modulating the donor-acceptor band gap, for instance, through the introduction of guest molecules, can profoundly influence their therapeutic efficiency.
(5) Recent advances in artificial intelligence (AI)-driven cancer immunotherapy [237,238] offer promising directions for combining porphyrin-based MOFs with AI. Such integration could open new pathways for diverse biomedical applications. AI can accelerate the directed evolution of porphyrin-based MOFs, thereby enhancing therapeutic efficiency.
(6) The presence of multiple physiological barriers, from systemic clearance to selective barriers such as the blood-brain barrier and complex tumor microenvironments, limits the therapeutic potential of nanomedicines in vivo [239]. To address these barriers, it is essential to develop smart porphyrin-based MOFs that are small in size, possess specific targeting and noninvasive diagnostic capabilities, and can activate treatments on demand.
(7) Last but not least, it is equally vital to develop multifunctional nanoplatforms by integrating porphyrin-based MOFs with other components or functional materials. For example, the incorporation of different metal nodes or contrast agents into porphyrin-based MOFs can enable fluorescence imaging, MRI-, computed tomography (CT)-, PAI-, or PTI-guided therapy.
(8) Translational challenges including scalability, reproducibility, and regulatory barriers should also receive growing attention. Addressing scalability issues requires the development of cost-effective and reliable manufacturing processes that maintain material performance. Reproducibility must be ensured through stringent synthesis protocols and advanced characterization techniques to guarantee consistent antibacterial efficacy across batches. Furthermore, early engagement with regulatory agencies is essential to establish clear safety guidelines and facilitate clinical approvals. Overcoming these multifaceted challenges will be critical to unlocking the full potential of porphyrin-based MOFs nanomaterials in real-world biomedical applications.

Drawing on current research, this work proposes key research priorities for multifunctional porphyrin-based MOFs toward biomedical applications. The structural tunability of porphyrin-based MOFs enables systematic investigation into the influence of steric configurations on photoelectrochemical performance. Moreover, by examining diverse metal active site architectures including single atoms, multimetal clusters, varied coordination geometries as well as steric modulation can significantly improve the therapeutic efficiency. Moreover, construction of multifunctional porphyrinic MOFs composites is also of great importance for practical application. Although significant challenges remain, the favorable biosafety profile at appropriate dosages and established therapeutic efficacy of these biomedicines illuminate promising pathways toward clinical translation. Ongoing research and innovation in this area will play a vital role in addressing pressing biomedicine issues.

## CRediT authorship contribution statement

**Tushuai Li:** Funding acquisition, Project administration, Visualization, Writing – original draft. **Wenxue Sun:** Data curation, Formal analysis, Investigation. **Hongying Pan:** Data curation, Formal analysis, Resources. **Qi Tang:** Software, Validation. **Lanxin Geng:** Software, Visualization. **Jin Liu:** Investigation, Methodology. **Guangfu Liao:** Conceptualization, Supervision, Visualization, Writing – review & editing. **Yanhua Zhang:** Funding acquisition, Project administration, Supervision, Writing – review & editing.

## Declaration of competing interest

The authors declare that they have no known competing financial interests or personal relationships that could have appeared to influence the work reported in this paper.

## Data Availability

Data will be made available on request.
